# News: Regions

**Published:** 2011-06

## Africa

### Vaccine contributes to dramatic reduction in meningitis in 3 African nations

Though Burkina-Faso, Mali and Niger form part of a belt across Africa traditionally associated with high rates of meningitis, statistics show that this year the rates of meningitis A are the lowest ever recorded during an epidemic season. This is the result of the successful introduction of a new vaccine MenAfriVacTM. Nearly 20 million people were vaccinated in a massive immunization campaign in these three countries. New analyses published in the journal *Health Affairs* shows that introducing this vaccine in seven highly endemic African countries could save as much as US$ 300 million (€ 210 million) over a decade and prevent about 1 million cases of disease. To bridge the funding gap of US$ 475 million (€ 332 million) for scaling up the programme, the GAVI Alliance has contributed US$ 100 million (€ 70 million) for the campaigns in Cameroon, Chad and Nigeria. Even though the vaccine is highly affordable at less than US$ 0.50 (€ 0.35) per dose, the significant funding gap needs to be closed if the vaccine is to be rolled out in all 25 countries of the meningitis belt by 2016.

### Investing in child health: promises of most African governments are not being kept

A report by the African Child Policy Forum that ranks the performance of 52 African governments on the basis of their spending in the key sectors that affect child welfare reveals that most countries allocated only between 4% and 6% of their national budget to health in 2008. This is well below their commitment to a target of 15%. The survey indicates that the governments of Tanzania, Mozambique, and Niger are the most committed in terms of using available resources for the well-being of children. Sudan ranks at the bottom, along with Guinea-Bissau, Eritrea, Burundi, Democratic Republic of Congo, Comoros, Sierra Leone, Angola and the Central African Republic. The report suggests they were ranked unfavourably because of low levels of their investments in sectors benefiting children. A declining trend of their allocations over the years and their relatively high military expenditure also contributed to low rankings.

### African Development Bank estimates that one in three Africans is now middle class

A recent report by the African Development Bank indicates that one in three Africans is now middle class. This rising group of consumers is expected to rival those of China and India. Record numbers of people in Africa own houses and cars, use mobile phones and the internet and send their children to private schools and foreign universities. The growth rate of the middle class over the past 30 years was about 3.1%, which is slightly faster than that of the total population. Tunisia, Morocco and Egypt had proportionately the largest middle classes in Africa, while Liberia, Burundi and Rwanda had the smallest. The African middle classes are more likely to have jobs with regular salaries or to own small businesses. They tend not to rely entirely on public health services and often seek more expensive medical care. The middle classes tend to have fewer children and spend more on their nutrition and schooling. However, the report also notes that poverty remains deeply entrenched, with more than half of Africa’s population living on less than US$ 2 (€ 1.4) a day.

### Africa Progress Panel: private sector is increasingly important for continents’ development

A report by the Africa Progress Panel indicates that public-private partnerships (PPPs) for development “are among the most promising, and potentially most effective” options for African growth, and that the private sector was playing an “increasingly important” role. “We have come to the conclusion that all actors (governments, private sector, civil society and international community) can do more to facilitate the spread of successful partnership models across countries and sectors – and that doing so is in their own self-interest,” says the report. However, the authors warned that too many companies were not adhering to the UN Global Compact when seeking to invest in the continent. Global Compact is a set of voluntary guiding principles aimed at businesses seeking to work in developing countries. The report also emphasizes that PPPs are not a “panacea for all of Africa’s problems.” Good governance and strong political leadership are the most important ingredients in African success. International donors also need to fulfil their financial commitments to the continent.

### Can mobile phones improve health across Africa?

Countries across sub-Saharan Africa are increasingly turning to cell phones to provide better health care to their population. The help sometimes comes in a form of a reminder text message to pick up a fresh batch of anti-HIV drugs in South Africa. On other occasions a dedicated network provides free voice calls or texts for consultations amongst physicians in Ghana. mHealth is revolutionizing medical practice in Africa and the value of the mHealth sector is estimated to up to US$ 60 billion, with mHealth companies on the rise. A recent report by the World Health Organization’s Global Observatory for eHealth indicates that in Africa, mobile penetration exceeds infrastructure development – including paved roads, and access to electricity and the internet.

## Asia

### Gates Foundation and Abu Dhabi crown prince plan immunization partnership in Asia

The Bill and Melinda Gates Foundation has announced a partnership with Sheikh Mohammed bin Zayed, Crown Prince of Abu Dhabi, through which they would be funding the immunization of about 40 million children in Afghanistan and Pakistan against deadly diseases such as polio, diphtheria, tetanus, whooping cough, pneumonia and hepatitis B. Bill Gates and Sheikh Mohammed bin Zayed have each pledged US$ 50 million (€ 46 million) towards the cause. The Gates Foundation has previously worked with UAE. In 2009 the two parties agreed to jointly fund Dubai Cares, a charity that works to improve the health and education of children in certain countries in the developing world.

### Midwives improve survival rates of mothers and infants in Afghanistan

Afghanistan has the one of the highest maternal and infant mortality rates in the world. It is estimates that approximately one of every 60 women dies giving birth, and 129 infants out of every 1000 die in their first year of life. However, this trend appears to be changing. The country’s infant mortality rate has been decreasing by an estimated 22% since 2003, thanks in part to better midwifery. As a result of multi-donor support – including from USAID, the European Commission, the World Bank, and the GAVI Alliance – the number of midwives in Afghanistan has increased from 467 in 2002 to more than 2500 in 2010. Though it appears there has been progress, the country’s health gains will be better appreciated when the USAID-supported Afghanistan Mortality Survey now under way is completed in summer 2011. The survey report will provide data on the magnitude of maternal mortality, the main causes of death, risk factors, and barriers that affect women’s access to care.

### Pakistan launches immunization drive against polio, targets 32 million children

The incidence rate of infective poliomyelitis has risen by 65% in Pakistan, partly as a consequence of floods in the summer of 2010 and conflicts in the northwest. The tribal belt has become a safe haven for the polio virus as vaccination has become impossible in many areas. Fighters have reportedly killed health workers and conservative tribal leaders have denounced the vaccine as “a part of a western conspiracy against their children.” Last year the tribal belt accounted for half of all infections in Pakistan. Worried now that the disease will spill into other polio-free parts of the country, the authorities in Pakistan launched an emergency drive to immunize 32 million children under the age of five against polio. This plan, supported by a US$ 65 (€ 42 billion) million grant from the Bill and Melinda Gates Foundation, should see armed police and paramilitary soldiers protecting teams of vaccinators in the most dangerous areas.

### Shortage of antiretroviral drugs in Myanmar and strategy for treating AIDS cases

While Myanmar’s AIDS crisis is not yet of the scale that some of the African countries are facing, the country lags far behind in its ability to deal with the spread of the epidemic. It is estimated that around 242 000 people (about 1% of the adult population) are infected with HIV, with as many as 120 000 currently needing antiretroviral treatment. But drugs are only available for 20 000 patients. The shortages mean that only those with a CD4 count of less than 200 will be considered eligible to receive antiretroviral medications from the aid organisations distributing these drugs. This strategy appears to be backfiring as the immune systems in these patients have completely collapsed, making them an easy target for tuberculosis and other infections.

### Infant deaths prompted Japan to suspend, then clear two childhood vaccines

Earlier this year Japan’s Health Ministry halted the use of Pfizer’s Prevenar and Sanofi’s ActHIB following the deaths of several children aged between 6 months and 2 years. Up to 6 children died shortly after receiving the vaccines, but no direct relationship could be established. Similar events have been reported sporadically from other countries in the recent past. In February 2010 three infants died after receiving Prevenar, but Dutch health authorities later said that they could not find any causal association between Prevenar and the deaths in vaccine recipients. Pfizer Inc. and Sanofi-Aventis SA said in March 2011 that Japanese health authorities cleared the way for the companies to resume sales of the two widely used childhood vaccines, whose use had been temporarily suspended amid safety concerns. A panel of medical experts soon concluded that the child deaths weren’t connected to the vaccines.

## Australia and Western Pacific

### The toll of Australia’s floods

The flooding that inundated much of south and central Queensland in January 2011 is the worst to hit Australia in half a century. It was triggered by unusually heavy monsoon rains just before Christmas and was fed by much higher than-normal rainfall ever since. More than 200 000 people were affected and the flood resulted in flnancial damages of more than US$ 20 billion (€ 14 billion). More than half of the country’s wheat crop was damaged, leading to 45% increase in global grain prices. The Australian parliament has approved the government’s plan to raise US$ 1.8 billion in floods tax to help rebuild the damaged infrastructure.

### Australia pledges additional US$ 140 million to global vaccination roll-out

At the GAVI Alliance conference in London in June 2011, Australia pledged to commit US$ 200 million (€ 139 million, ie, an extra US$ 140 million (€ 97 million), during the next two years to support a vaccination program to help fight infant mortality in the world’s poorest countries. This commitment comes at a time when GAVI was worried about facing a substantial shortfall in its funding needs. The move makes Australia the sixth largest contributor to GAVI in direct funding. The donation will help support introduction of a rotavirus vaccination programme in African and Asian countries and will help vaccinate an extra 7.1 million children globally.

### Rotavirus vaccination proves consistently effective throughout the world

New data which were published in *Journal of Pediatrics and Child Health* in May 2011, and which prominently involve Australia, indicate that in the years 2007 and 2008 hospitalisations for rotavirus gastroenteritis declined by 75%. This dramatic fall comes after vaccine introduction, and it is expressed in comparison to the mean annual hospitalisations during six years prior to vaccine introduction (ie, during the period between 2001 and 2006). The greatest decline was seen in those younger than 12 months (93%), but the reduction occurred consistently across all age groups. The reduction was even recorded among children who were not eligible for immunisation, suggesting an effect on herd immunity. Similar effects have been shown in studies conducted in other countries (United States, El Salvador, Belgium), where hospitalizations and clinic visits for rotavirus diarrhoea have declined substantially. The decline typically ranges from 60% to over 90%.

### New family planning initiatives supported by AusAID

Data released by the Australian government have shown an intention to spend around 20 million Australian dollars in 2010 and 2011 on family planning activities in developing countries. The aid program should support improved health outcomes for women. Health programs would target women in Indonesia, Papua New Guinea, Bangladesh, Pakistan, Cambodia, Solomon Islands, Afghanistan, East Timor and Nepal. The aid program will also be supported by UNFPA, International Planned Parenthood Federation, UNICEF and the GAVI Alliance. The key activities will include family planning information and education, establishing safe birth sites, and supplying and distributing contraceptives.

### Australian Medical Association records the first fatal diphtheria case in nearly two decades

A 22-year-old Brisbane woman died in hospital in April 2011 after contracting diphtheria from a friend who had returned from overseas. This caused both surprise and concern in the medical community in Australia, because the disease is virtually unheard of there. Experts believe that the woman wasn’t immunized. Queensland Health said it last confirmed a case of diphtheria in the state in 1993. The vice president of the Australian Medical Association, Steve Hambleton, said he had never heard of a case in Australia during his work as a health professional. The vaccination coverage for diphtheria in Australia is more than 90%.

## China

### China reveals its foreign aid policy plans

In April 2011, the Chinese government published its first report – referred to as the “white paper” – on foreign aid. The report claims that China’s foreign aid to Africa was motivated by solidarity. China’s budgeted foreign aid grew steadily (by nearly 30% a year) between 2004 and 2009, with more than 40% of this funding spent on grants. The report does not provide more detailed information on aid flows, though. It has been suggested in some media that Beijing withheld details of its foreign assistance programme to prevent domestic criticism. Despite its remarkable growth, China is still home to the world’s second largest population of poor people, with more than 200 million still living on less than US$ 1.25 (€ 0.9) per day.

### China increases its spending on health

China plans to increase its health spending by 16.3% in 2011, to approximately US$ 26 billion (€ 18 billion) in total. The government will increase its funding for basic health services to 25 yuan (US$ 3.8, € 2.6) per capita, which is a 67% increase from last year. Health insurance subsidies are also expected to grow from US$ 18.2 (120 yuan, € 12.6) to US$ 30.4 (200 yuan, € 21.1) per person. This increase in health spending is a major part of China’s plan to create affordable universal access to medical treatment, hospital care and pharmaceuticals. By lowering health-related costs, the government also hopes to drive domestic consumption. Increasing the capacity of health facilities and health-insurance coverage in rural China is also a part of the funding target.

### Disability care volunteers to double in China by 2015

The China Disabled Persons’ Federation (CDPF) reported that the government hopes to double the number of active disability care volunteers in China by the year of 2015. This is the latest effort to aid disabled people over the next five years. China currently has about 83 million disabled people. Only a minority of them have access to ‘one-on-one’ aid services, which are provided by up to 5.3 million registered volunteers throughout China. The authorities will aim to gradually recruit a further 5 million volunteers to aid disabled people and assist them in leading a valuable and dignified life. Lu Shiming, the vice chairman of the CDPF, stated that the country has made much progress in providing social security for the disabled.

### China set to ban smoking in public places

China is set to ban smoking in public places including restaurants, bars and buses. Previously, China had only banned it in hospitals. The new regulations came into effect on the first day of May 2011, as announced by the Chinese Ministry of Health. The new regulations also ban cigarette vending machines in public areas and call for health education programmes to warn about the dangers of smoking. China has more than 300 million smokers – almost a quarter of its population. It is thought that smoking-related illnesses kill more than 1 million people every year. The measures of enforcement and the punishment for breaking the rules have yet to be announced in the new regulations. Smoking is still permitted in workplaces.

### China launches orphan welfare database

In March 2011 China launched a nation-wide database to compile information on orphans and ensure welfare services. It will record data on the country’s estimated 712 000 orphans, including basic identification details and photographs. This should provide precise statistics on orphans across the country. According to the Ministry of Civil Affairs, the database is especially designed to ensure the timely payment of basic living expenses for orphans and to monitor the distribution of living allowances paid to orphans, in order to maintain accuracy and fairness in welfare coverage.

## Europe

### Lethal *Escherichia coli* strain’s outbreak concerns experts

The last week of May 2011 saw the outbreak of a toxic strain of *Escherichia coli* bacteria, which has so far left more than 2000 people ill and claimed at least 17 lives across Western Europe and the United States of America. Experts believe that the outbreak, which has its origin in Germany, may be caused by the deadliest *E coli* strain ever recorded. Though the exact source of the illness still remains poorly understood, it was generally felt that eating contaminated vegetables and salads may have resulted in the infection. None of the currently available antibiotics were effective against this strain. Some US experts expressed the opinion that antibiotics should not even be used to treat this particular infection.

### Large peaks in measles cases across Europe

There has been a 10-fold rise in measles cases this year in England and Wales. The data released by the Health Protection Agency (HPA) revealed 334 confirmed cases of measles for the period January to April 2011, compared with 33 in the same period last year. The highest number of cases was recorded among under-25-year olds who were not vaccinated. Other European countries like France, Spain, Former Yugoslav Republic of Macedonia, Serbia and Turkey have also seen a similar sharp increase in measles cases, with France being the worst hit of them all. This information has triggered concerted efforts by health agencies across Europe to improve measles vaccination rates.

### United Kingdom will commit an extra 814 million pounds to buying vaccines for the poor

Despite a weak economy and cuts in public spending, the UK Prime Minister David Cameron pledged GBP 814 million (US$ 1303 million, € 906 million) at a GAVI Alliance conference in London in June 2011 to help vaccinate children around the world. The purchased vaccines will protect children against preventable infectious diseases, such as pneumonia. This move will see UK’s total contribution increased to GBP 1.5 billion pounds, which makes it the GAVI Alliance’s largest donor. Britain’s total contribution is now about five times larger than the amount pledged by the United States, more than 30 times higher than Germany’s € 49 million (GBP 44 million, US$ 70 million), and almost 50 times larger than Spain’s donation. According to GAVI, this investment could help save the lives of four million children over the next four years.

### Russia spent US$ 472 million on foreign aid in 2010

Russia has reportedly spent US$ 472 million (€ 328 million) in 2010 on foreign aid. This reinforced the position of the large country as an important aid donor, rather than a recipient. Of this, more than US$ 80 million (€ 56 million) was spent on improving health care in developing countries. The Russian government is considering setting up its own aid agency in a bid to increase its influence in the developing world. Some analysts think that the intention behind the move is to match the growing influence of Brazil and South Africa in the developing world. Russia has admitted that a more consistent policy on international development would help strengthen its international position. It is also expected to spur Russia’s domestic development by promoting trade and economic cooperation with countries that receive Russian aid.

### Growing number of European academic institutions focused on global health

A new organisation – the European Academic Global Health Alliance (EAGHA) – brought together a large number of academic institutions in Europe whose activities address the broad scope of global health. It was launched in early 2011. Its main aims are to create a forum for European academic institutions interested in global health, exchange views and ideas, develop a European voice on global health issues, influence relevant policies and bring together international health, tropical medicine and public health institutions. EAGHA has already attracted 36 member institutions from 19 countries, and will be hosted by the Association of Schools of Public Health in the European Region (ASPHER) in Brussels. It has also developed partnerships with institutions in more than 50 low and middle income countries

## India

### Indian economy is growing, but its children are still under-nourished

A study by Harvard School of Public Health (HSPH) revealed that India’s impressive economic growth has not led to a reduction in under-nutrition among its children. The study analysed economic and children’s growth patterns from databased on the National Family Health Surveys on 77 326 Indian children in the periods 1992–1993, 1998–1999 and 2005–2006. Under-nutrition was worst in the poor and populous states like Bihar, Madhya Pradesh and Uttar Pradesh, in comparison to the northeastern and southern states. While the researchers found the prevalence of under-nutrition slightly decreasing during the study period, the decline did not correspond to the increase in economic growth. The authors concluded that India is not on track for achieving the target for Millennium Development Goal (MDG) of reducing child mortality.

### Rotavirus vaccines launched in India

In March 2011, MSD (a fully owned subsidiary of Merck & Co.) launched the rotavirus vaccine – RotaTeq – in India. RotaTeq is a pentavalent rotavirus vaccine that helps in preventing rotavirus associated gastroenteritis, estimated to be associated with about 39% of all diarrhoea-related hospital admissions in India among children aged less than five years. Since this vaccine was priced beyond the reach of the common people, there was considerable interest in the products being developed by local Indian companies. One of the manufacturers, Bharat Biotech, has announced that it plans to sell Rotavac, India’s first indigenously developed Rotavirus vaccine, to global public markets at a price of US$ 1 (€ 0.7) per dose. The company is not just targeting Indian markets: they are well positioned to manufacture and supply the vaccine to national immunization programmes across the world.

### All Indian newborns will get hepatitis B vaccine

The World Health Organization estimates that there are over 40 million Hepatitis B surface antigen (HBsAg) carriers in India and that every year over 100 000 Indians die from illnesses related to HBV infection. It is estimated that of the 25 million infants born in India every year, over 1 million run the lifetime risk of developing chronic hepatitis B virus (HBV) infection. In November 2010, the Indian Ministry of Health decided to introduce hepatitis B vaccination for all newborns as part of the national immunization programme (NIP) from April 2011. Hitherto, hepatitis B vaccination (initiated as a pilot in the year 2002 with GAVI Alliance support), was only being carried out in 10 states with relatively better child health indicators. Now, as a result of this far-reaching decision, all newborns in India will be immunised after birth and then at six, 10 and 14 weeks.

### A potential anti-tuberculosis agent is fished out of the sea

From a coral reef off Rameswaram in South India, Indian scientists have isolated Transitmycin, a novel antibiotic that could fight tuberculosis and HIV better than most drugs available today. Unlike many of the present anti-tuberculosis drugs, transitmycin acts on dormant bacilli, which are a reservoir of infection. Doctors also found this compound to be active against HIV (subtypes B and C). While the project has so far cost US$ 50 000 (€ 34 699), it may take another US$ 700 million (€ 486 million) to develop the drug. Scientists estimate that it would take another 10 years before the drug reaches the market, but are hopeful that this could be an entirely novel and important medicine.

### Two thirds of India’s population could be middle class within 15 years

A report by Asian Development Bank indicates that nearly 70% of India’s population could be middle class within 15 years if the country’s economy continues to show sustained growth. If Asian middle class consumers can substitute for those in advanced economies, the Asian countries will gradually become major exporters to each other, without excessive reliance on the consumer markets of Europe or North America. The Indian economy, Asia’s third-largest, has steadily been growing above 8% annually and it has contributed significantly to rising prosperity. The report says that Asia’s rise will be led by China, India, Indonesia, Japan, South Korea, Malaysia and Thailand. By 2050, those seven economies alone should account for 45% of global GDP, and this may be a conservative estimate.

## The Americas

### United States will provide US$ 450 million for vaccines to save 4 million children’s lives

At the GAVI conference in London, the United States government pledged to commit US$ 450 million (€ 312 million) over the next three years to the Alliance. The funding will provide vaccines for more than 250 million children, preventing 4 million premature childhood deaths and a large amount of suffering from diseases like pneumonia and diarrhoea. Although this is much lower than the funds committed by some other countries, USAID chief Raj Shah said: “In this fiscal climate, a multi-year pledge is an extremely difficult commitment to make. But we have made tough reallocations across our portfolio in order to make that commitment because only a multi-year pledge will ensure the highest possible return for every taxpayer dollar.”

### Stephen Harper moves to Geneva to keep track of US$ 40 billion fund for women’s health

In January 2011, the Canadian Prime Minister Stephen Harper co-chaired a meeting of a United Nations’ commission in Geneva. The task of the commission will be keeping track of how US$ 40 billion (€ 28 billion) is being spent on improving the health of women and children in poor nations.

### Re-introduction of controversial US bill would hurt world’s poorest women

Republicans in the US House of Representatives are pushing to re-introduce a law that would not only hurt women’s health and reproductive rights in the developing world, but also affect some of the poorest and most vulnerable women in the United States. The bill includes cutting funding to the ‘Title X’ family-planning program, which would affect low-income families’ access to contraception and sexual health services in the United States. In an international context, the bill seeks to re-impose the ‘global gag’ rule which strips international non-governmental organizations of all funding if they convey any information about abortion, along with other aspects of family planning, such as contraception and HIV/AIDS prevention, to the communities which they serve.

### Prevention of cholera deaths in Haiti

In the absence of new interventions, it is estimated that the number of Cholera cases in Haiti may rise up to 800 000 this year, resulting in more than 11 000 deaths. These estimates, published in *The Lancet* in May 2011, are substantially higher than those generated by UN agencies. The authors point out that with an extremely weak sanitary infrastructure and a favourable environment for indefinite persistence, cholera is likely to remain a threat in Haiti for many years. They conclude that a combination of three approaches (access to safe drinking water, provision of vaccines, and improved case management using oral rehydration and antibiotics) are needed to have a beneficial effect in a post-disaster situation.

### More than 100 cholera cases recorded in Venezuela

In January 2011, Venezuela reported 111 cases of cholera. It is thought that these cases became infected after consuming contaminated food at a wedding in the Dominican Republic. Lobsters bought from a town adjoining cholera-stricken Haiti have been identified as the likely source of infection. Before this, the last reported case of cholera in Venezuela was recorded more than 10 years ago. The fear of cholera has lead to the deportation of Haitian immigrants since the beginning of the year. The Dominican Republic has acknowledged only one cholera death since the outbreak began in Haiti.

